# Author's Reply to: Periodic Manual Algorithm Updates and Generalizability: A Developer’s Response. Comment on “Evaluation of Four Artificial Intelligence–Assisted Self-Diagnosis Apps on Three Diagnoses: Two-Year Follow-Up Study”

**DOI:** 10.2196/29336

**Published:** 2021-06-16

**Authors:** Aleksandar Ćirković

**Keywords:** artificial intelligence, machine learning, mobile apps, medical diagnosis, mHealth, symptom assessment

I would like to thank Gilbert et al [1] for their useful contributions and tips to help improve our work [2].

Regarding their first point of critique, it is true that the evaluation lacked the technique to determine with certainty whether the algorithms used were or were not “locked” according to the terminology used by the Food and Drug Administration. For that reason, I stated in the *Discussion* section that some details of the study results only *suggest* a nonlocked direction [2]. Unfortunately, the abstract lacks this softening term. It remains true, however, that for regulators and physicians to correctly assess the functions of such software, either the algorithms would have to be disclosed—which might not be in the interest of the competing companies—or advanced testing models would have to be developed, possibly leading to a “cat-and-mouse” game similar to other regulatory fields. In developed countries where legal liabilities have to be clearly distributed and delimited, neither responsible physicians nor authorities will want to rely on mere published statements of company spokespeople alone—hard data from unbiased sources will be needed in the future.

The subsequent publication of the Ada study of a 200-vignette assessment is highly appreciated, as herein the authors were able to evaluate a considerably larger amount of data [3]. As I had stated, with a small sample size as a limitation, it does remain a possibility that the apps’ poor results as demonstrated in our study are within data variance norms or due to our own bias. However, the results did capture an experience that any potential user or patient with eye problems could have possibly encountered in the same way. Regulations will hopefully minimize risks for all users. Only thorough investigations, including but not limited to manufacturers’ evaluations, will help us better understand the effects the apps will have on public health. Until then, our knowledge base will consist of various analyses with possibly conflicting results that we will have to make sense of.
